# Molecular-based tumour subtypes of canine mammary carcinomas assessed by immunohistochemistry

**DOI:** 10.1186/1746-6148-6-5

**Published:** 2010-01-28

**Authors:** Francesco Sassi, Cinzia Benazzi, Gastone Castellani, Giuseppe Sarli

**Affiliations:** 1 Department of Veterinary Public Health and Animal Pathology - Division of Veterinary Pathology, Faculty of Veterinary Medicine, University of Bologna, Via Tolara di Sopra, 50 - 40064 - Ozzano dell'Emilia (Bologna), Italy; 2 Department of Veterinary Morphophysiology and Animal Production, Faculty of Veterinary Medicine, University of Bologna, via Tolara di Sopra 50-40064 Ozzano Emilia - Bologna - Italy

## Abstract

**Background:**

Human breast cancer is classified by gene expression profile into subtypes consisting of two hormone (oestrogen and/or progesterone) receptor-positive types (luminal-like A and luminal-like B) and three hormone receptor-negative types [human epidermal growth factor receptor 2-expressing, basal-like, and unclassified ("normal-like")]. Immunohistochemical surrogate panels are also proposed to potentially identify the molecular-based groups. The present study aimed to apply an immunohistochemical panel (anti-ER, -PR, -ERB-B2, -CK 5/6 and -CK14) in a series of canine malignant mammary tumours to verify the molecular-based classification, its correlation with invasion and grade, and its use as a prognostic aid in veterinary practice.

**Results:**

Thirty-five tumours with luminal pattern (ER+ and PR+) were subgrouped into 13 A type and 22 B type, if ERB-B2 positive or negative. Most luminal-like A and basal-like tumours were grade 1 carcinomas, while the percentage of luminal B tumours was higher in grades 2 and 3 (Pearson Chi-square P = 0.009). No difference in the percentage of molecular subtypes was found between simple and complex/mixed carcinomas (Pearson Chi-square P = 0.47). No significant results were obtained by survival analysis, even if basal-like tumours had a more favourable prognosis than luminal-like lesions.

**Conclusion:**

The panel of antibodies identified only three tumour groups (luminal-like A and B, and basal-like) in the dog. Even though canine mammary tumours may be a model of human breast cancer, the existence of the same carcinoma molecular subtypes in women awaits confirmation. Canine mammary carcinomas show high molecular heterogeneity, which would benefit from a classification based on molecular differences. Stage and grade showed independent associations with survival in the multivariate regression, while molecular subtype grouping and histological type did not show associations. This suggests that caution should be used when applying this classification to the dog, in which invasion and grade supply the most important prognostic information.

## Background

Human breast cancer is considered a heterogeneous disease, and is classified by gene expression profile into subtypes consisting of two hormone (oestrogen and/or progesterone) receptor-positive types (luminal-like A and luminal-like B) and three hormone receptor-negative types [human epidermal growth factor receptor 2-expressing, basal-like, and unclassified ("normal-like") ] [1-3]. Epidermal growth factor receptor 2 is indicated in different ways in the literature, namely HER2, c-ERB-2, neu, ERB-B2. This study uses the acronym ERB-B2. Follow-up studies have shown these subtypes to be conserved across diverse patient series and array platforms [4,5], and that different gene expression-based predictors are likely tracking a similar common set of biological subtypes, with significant agreement in predicting patients' clinical outcome [6].

**Figure 1:**
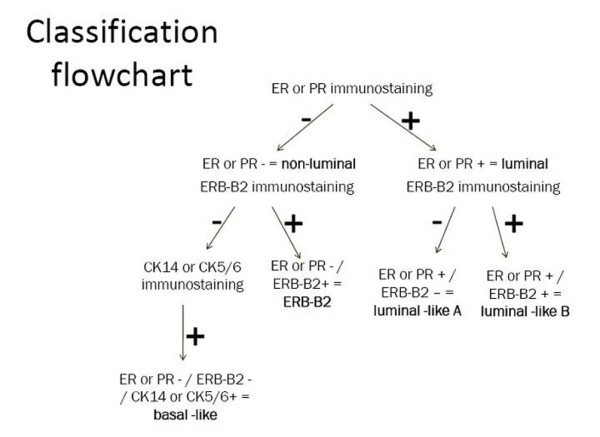
**Classification flowchart followed in the present study (from Conforti et al. **[7]** modified)**.

Cost and complexity issues have to date rendered gene expression profiling impractical in laboratories equipped for routine diagnostic tests. However, some of the immunohistochemistry surrogate panels proposed can potentially identify the molecular-based groups according to classification flowcharts [7] (figure 1). The panels encompass at least anti-oestrogen receptor (ER), anti-progesterone receptor (PR), anti-ERB-B2 and anti-basal cytokeratin antibodies (CK 5/6 and 14) [8,9]. Basal-like breast cancers are characterized by the lack of ER, PR and ERB-B2 expression and cytokeratin 5/6 and/or epidermal growth factor receptor expression [7], whereas the luminal-like subtype is characterized by ER or PR expression [7] and further subgrouped into luminal A or B depending on the absence or presence of ERB-B2 expression [10]. Negativity for luminal biomarkers, positivity or negativity for basal markers and ERB-B2 positivity characterizes the ERB-B2 overexpressing group [10].

Canine mammary tumours are considered a spontaneous animal model of human breast cancer [11,12] prevalently on a histomorphological basis, but several differences between the tumours of the two species must be taken into account to make any comparisons feasible. Myoepithelial cell proliferation is a frequent finding in the so-called complex and mixed patterns [13,14], but it is an uncommon feature of breast cancer in women [15]. Multiple tumours at the first clinical presentation are very common in the female dog [16,17], but not in women [15]. Do these differences indicate species-specific tumour genesis and development? The molecular-based classification, recently adopted for breast cancer, seems to be a better tool than morphological features to establish objective similarities between the two models. In canine mammary tumours, the loss of hormone receptors in tumour progression is known [18,19], as is the overexpression of ERB-B2 products in malignancies [20,21]. However, to our knowledge, only one recent investigation merged these two features to devise a molecular-based subgrouping of malignant tumours [22]. This study applied the molecular classification to a series of canine mammary carcinomas, using a panel of antibodies apt to demonstrate a role in prognosis and identification of molecular groups similar to human breast cancer.

The present study aimed to apply an immunohistochemical panel (anti-ER, -PR, -ERB-B2, -CK 5/6 and -CK14) in a series of canine malignant mammary tumours to verify the molecular-based classification, its correlation with invasion and grade, and its use as a prognostic aid in veterinary practice.

## Methods

### Cases and follow-up data

Forty-five tissue samples were obtained from female dogs with malignant primary mammary tumours. All the dogs underwent surgery at the University Clinic of Veterinary Surgery (Bologna, Italy) and in private clinics. The tissue samples were immediately fixed in 10% buffered formalin and routinely processed. Before surgery and every 2 mo over a 2-yr period, radiographs of the thorax and ultrasonograms of the spleen, liver, and kidneys were obtained. None of the dogs had distant metastases (M0) at surgery. Follow-up data were collected over this period and expressed as survival time (the time between surgery and death due to the tumour, either spontaneous death or euthanasia). After 24 mo of follow-up, the animals still alive were considered assessed and those that had died non-assessed.

### Tumour grade and invasion

Histologic diagnoses were achieved on hematoxylin and eosin stained slides according to Misdorp et al. [13], and tumour grade according to Lagadic and Estrada [23]. Invasiveness (stage) was determined following a previously proposed system [24]: stage 0 = tumours without stromal invasion (*in situ*); stage I = tumours with stromal invasion; and stage II = tumours with neoplastic emboli in vessels.

### Immunohistochemistry

For each tumour, five 4-micron-thick consecutive sections were used for anti-ER, -PR, -ERB-B2, -CK5/6 and -CK14 antibodies. Sections were dewaxed in toluene and rehydrated. Endogenous peroxidase was blocked by immersion in 0.3% hydrogen peroxide for 20 min. Sections were then rinsed in Tris Buffer and antigen was retrieved with citrate buffer (2.1 g citric acid monohydrate/litre distilled water), pH 6.0, and heating for two 5 min periods in a microwave oven at 750 W, followed by cooling at room temperature for 20 min. The primary antibodies are summarized in Table 1. All antibodies were incubated with the tissue sections overnight at 4°C, except for anti-PR incubated 1.5 hours at 37°C, and were followed by a commercial streptoavidin-biotin-peroxidase technique (LSAB Kit, Dako, Amsterdam, The Netherlands). Diaminobenzidine (0.05% for 10 min at room temperature) was used as chromogen. Slides were counterstained with Papanicolaou's hematoxylin. Negative controls were obtained substituting the primary antibody with an unrelated monoclonal antibody of the same isotype. As positive controls to assess the cross-reactivity with canine tissues and the specificity of the immunohistochemical stain, sections of canine normal mammary gland (anti-ER, -PR and -CK14 antibodies), canine skin (anti-CK5/6) and bovine pancreas (anti-ERB-B2 antibody) were used following the same protocols.

**Table 1:** Primary antibodies used for immunohistochemistry in the current study.

Antibody (anti-)		Clone	Manufacturer	Working concentration
Oestrogen Receptor	polyclonal		Zymed (South San Francisco, Ca)	1:50

Progesterone Receptor	monoclonal	PR 4-12	EMD Biosciences (San Diego, Ca)	1:10

ERB-B2	polyclonal		Dako (Glostrup, Denmark)	1:50

Cytokeratin 14	monoclonal	Ab-1 (LL002)	NeoMarkers (Fremont, Ca)	1:300

Cytokeratins 5/6	monoclonal	D5/16B4	Zymed (South San Francisco, Ca)	1:100

### Interpretation of immunohistochemical staining

Staining data were classified semiquantitatively. All immunohistochemical markers were accessed as negative and positive groups. Specifically, negative cases were those that displayed no staining or staining in less than a certain percentage of positive tumour cells, and positive cases were those with unequivocal staining in at least a certain percentage of tumour cells. Positivity for CK5/6 and CK14, according to Kim et al. [9] was defined as the detection of at least 1% of invasive tumour cells showing strong cytoplasmic staining. Immunostaining for ERB-B2 was interpreted as positive when at least 10% tumour cells showed moderate to strong complete membranous staining [25]. Cases were considered positive for ER or PR when nuclear staining was observed in at least 5% tumour cells [19].

### Grouping molecular subtypes

Based on a modified classification of Yang et al. [10], tumour subtypes were defined as luminal-like A (ER+ and/or PR+, ERB-B2 -, any CK5/6 or CK14), luminal-like B (ER+ and/or PR+, ERB-B2 +, any CK5/6 or CK14); ERB-B2-expressing (ER-, PR-, ERB-B2 +, any CK5/6 or CK14); basal-like (ER- and PR-, ERB-B2 -, CK5/6 + and/or CK14+), and unclassified or normal-like (negative for all markers).

### Statistical analysis

Pearson chi-squared statistic was used to test the association of the molecular subtypes with numerosity of cases homogeneous for invasion (0, I, II), grade (1, 2, 3) and histotype group. Histotype grouping was as follows: 1) simple: including all the simple carcinomas; 2) complex/mixed: comprising all the complex carcinomas and carcinomas in mixed tumours; 3) others: including the other histological types considered in the Misdorp et al. [13] classification system. Kaplan-Meier survival curves were calculated. To test the influence of molecular-based subgrouping (luminal-like A vs luminal-like B vs basal-like), stage (0 vs I vs II) and grade (1 vs 2 vs 3 separately for cases with and without vascular invasion) on survival, comparisons were verified by logrank test corrected for multiple comparisons. To investigate the simultaneous influence on survival, variables such as molecular-based subgrouping (luminal-like A vs luminal-like B vs basal-like), stage (0 vs I vs II), grade (1 vs 2 vs 3) and histotype (simple vs complex/mixed) were also examined by multivariate regression with the proportional hazard Cox regression model for censored data. Analyses were performed by CSS software (Statsoft, Tulsa, OK) statistics, and a conventional 5% level was used to define statistical significance.

## Results

The 45 female dogs ranged from four to 15 years of age (mean ± standard deviation = 9.66 ± 2.36; median = 10). Twenty-seven were crossbred and 17 purebred, namely six German shepherds, five Yorkshire terriers, two pointers, two dachshunds, two poodles. For one remaining case breed and age were not known.

Histologic diagnoses included seven *in situ *carcinomas, 19 simple carcinomas (11 tubulo-papillary and six solid types), 25 complex/mixed carcinomas (16 complex tubulo-papillary type and four carcinomas in mixed tumours) and one squamous carcinoma (included in the "other" group). Staging comprised seven non-infiltrating tumours (*in situ *carcinomas, stage 0 i.e. the same identified by the morphological classification system), 24 carcinomas with stromal invasion (stage I), and 14 carcinomas with vascular emboli and/or regional lymph node involvement (stage II). Grade assessment revealed 18 grade 1 (16 without and two with vascular invasion), 14 grade 2 (11 without and three with vascular invasion) and 13 grade 3 (four without and nine with vascular invasion) tumours. At 24 months after mastectomy, 33 of 45 cases were still alive and 12 cases had died, either euthanized or spontaneously, in both cases due to tumour spread.

Following the score criteria, 35 cases were ER and/or PR positive whereas ten were negative; 22 cases were classified positive to ERB-B2 and ten positive for basal cytokeratin expression.

**Figure 2:**
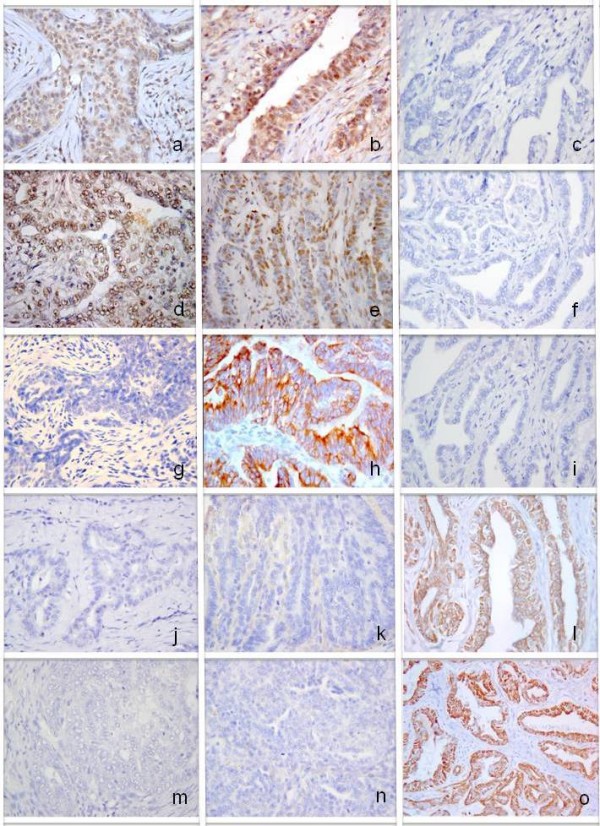
**Immunohistochemical expression of the panel of antibodies applied by IHC in canine mammary carcinoma**. **a**-**c **PR staining; **d**-**f **ER staining; **g**-**i **ERB-B2 staining;**j**-**l **CK 14 staining; **m**-**o **CK 5/6 staining. Each column refers to a distinct molecular subtype. From left to right, each column represents luminal A, luminal B and basal subtypes. 400×.

According to the criteria adopted for molecular subtype grouping, luminal B cases (n = 22 cases) accounted for 49% of the tumours, followed by luminal A (n = 13, 29%), and basal-like (n = 10, 22%). No case was ERB-B2 expressing and no case was unassessed. Immunohistochemical expression of the panel of antibodies in the different subtypes is reported in figure 2.

No difference was present in the percentage of molecular subtypes among the three invasion groups (Pearson Chi-square P = 0.76). A higher percentage of luminal A and basal cases was recorded among grade 1 carcinomas (luminal A 50%, luminal B 12%, basal 38%). The percentage of luminal B cases was higher in grade 2 (luminal A 14%, luminal B 63%, basal 23%) and grade 3 (luminal A 15%, luminal B 77%, basal 8%) carcinomas (Pearson Chi-square P = 0.009). No difference was found in the percentage of molecular subtypes between simple and complex/mixed carcinomas (Pearson Chi-square P = 0.47). The group indicated as "others" was not investigated because of the paucity of cases (one squamous carcinoma). These results are summarized in table 2.

**Table 2:** Pearson Chi squared statistic.

	Luminal-like A (n. 13)	Luminal-like B (n. 22)	Basal-like (n.10)	P value
Invasion (stage)				0.76
Non-infiltrating (0)	1	4	2	
Stromal invasion (I)	8	10	6	
Vascular invasion (II)	4	8	2	
Grade				0.009
Grade 1	9	3	6	
Grade 2	2	9	3	
Grade 3	2	10	1	
Histotype				0.47
Simple	6	10	3	
Complex/mixed	6	12	7	
Other	1	0	0	

Comparison between molecular phenotypes (luminal-like A, B, and basal-like) and invasion or grade or histotype groups.

**Figure 3:**
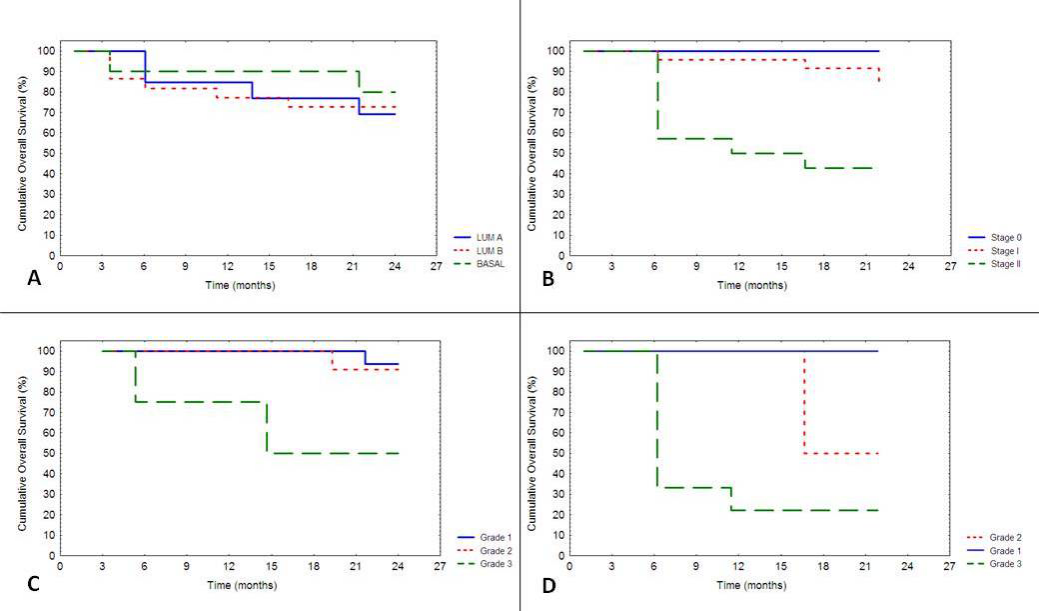
**Kaplan-Meier overall survival curves of the different molecular (A), stage (B) and grade groups**. The grade curves are presented separately for cases with (C) or without (D) vascular invasion.

Luminal-like A, luminal-like B and basal-like groups revealed no difference by survival analysis (P = 0.85), but basal-like tumours showed a better outcome than the luminal groups, and luminal B group had a slightly worse outcome than luminal A cases (figure 3A). Invasion (P = 0.0025, figure 3B) and grade, separately for tumours with (P = 0.047, figure 3C) and without (P = 0.03, figure 3D) vascular invasion, were significantly associated with prognosis.

Stage and grade showed independent associations with survival in the multivariate regression (table 3), while molecular subtype grouping and histological type did not show associations.

**Table 3:** Multivariate regression

Variable	Beta*	standard error	hazard ratio*
Stage			
Stage 0	1.32 (0.05/2.59)	0.65	3.76 (1.05/13.38)
Stage I			
Stage II			

Grade			
Grade 1	1.25 (0.29/2.21)	0.49	3.50 (1.34/9,12)
Grade 2			
Grade 3			

Histological type			
Simple	-0.72 (-1.97/0.53)	0.64	0.48 (0.14/1.71)
Complex/mixed			

Molecular group			
Luminal-like A	-0.31 (-1.43/0.81)	0.57	0.73 (0.24/2.24)
Luminal-like B			
Basal-like			

Proportional hazard Cox regression Chi^2 ^= 20.20 - df = 4 - P = 0.00046. Groups are indicated for each variable. Only stage and grade show a higher coefficient of regression (beta) than their standard error, thereby attributing them an independent prognostic significance.*(low/up) 95% confidence interval.

## Discussion

Human breast cancer is considered a heterogeneous disease according to oestrogen receptor, tumour grade and age [26]. Studies examining comprehensive gene-expression patterns using hierarchical clustering disclosed four clusters and suggested a four-way classification of breast cancer: luminal-like (subsequently sub-classified as A and B types), basal-like, HER-positive and normal-like [1,3]. The main goal of applying this innovative classification system is to demonstrate its role in prognosis. In addition, this molecular-based classification of breast cancers provides an opportunity to investigate biological questions in homogeneous entities, and to enrich a specific subclass with a relevant signal. This might prove superior to studies in a "diluted" population enrolling breast cancer patients without distinction [27].

However, molecular-based sub-grouping according to gene expression profile is impractical in laboratories equipped for routine diagnostic tests, and provides an imprecise picture of the actual sub-classes [27] because mRNA levels do not always correspond to changes in protein expression [9]. The protein quality depends not only on the amount and rate of transcription and translation, but also on degradation and rate of transport from cells. In addition, tissues used for mRNA profiling include both tumour and stromal cells. Therefore, immunohistochemistry may be beneficial in identifying the status of cellular expression and specific location of the protein [9]. Immunohistochemical-based markers have been proposed to help identify the molecular categories and define these sub-classes [9,10,7,8].

In veterinary medicine, mammary gland tumours are classified morphologically providing good prognostic indications [28,29] that may be enhanced by further prognostic tools such as staging [24], histological grade [23], proliferation indexes [30,31], hormone receptor status [19,32] and adhesion molecules expression [33,34]. However, canine malignancies form a heterogeneous group of different molecular driven tumours which may benefit from a classification that takes such molecular differences into account.

The present study applied a panel of antibodies commonly used to characterize the molecular groups in human pathology, and identified three tumour groups (luminal-like A and B and basal-like) out of the five known groups (no ERB-B2 or normal-like cases were present in our study, due to either the paucity of cases or to the epidemiological situation). Gama et al. [34], in a series of 102 canine mammary carcinomas, identified four tumour groups (luminal-like A and B, basal-like, and HER2 overexpressing). Investigations in veterinary medicine seem to confirm the importance of a molecular characterization of canine mammary tumours, but further work is necessary to confirm the data from the present study and that of Gama et al. [34].

Our study showed that the molecular-based classification of canine mammary cancer was related to grade, but not to invasion and morphologic classification. Luminal-like A group tumours included a significantly higher percentage of grade 1 tumours than luminal-like B group in which grades 2 and 3 cancers prevailed. Gama et al. [34] found that only the basal-like tumour group was associated with grade and the presence of vascular invasion. The molecular-based subgrouping of human breast cancer was associated with histological grade results in several investigations [10,9,7]. Simple or complex/mixed patterns do not seem to be correlated to a specific molecular group in our study, whereas Gama et al. [34] found the complex types associated with luminal-like A tumours, and simple pattern and carcinosarcomas to HER2 overexpressing and basal-like groups.

Application of this classification system in human medicine has yielded conflicting results on the relation with different clinico-pathological variables and survival where only the ERB-B2 overexpressing [9] or only the basal-like [2] tumours showed evidence of a significantly shorter survival. Our investigation failed to disclose any association between the molecular-based classification system and survival, whereas Gama et al. [34] found only the basal-like group significantly associated with a short survival. The difference between these studies can be explained by the different panels and criteria adopted to define the positivity to basal markers (cytokeratin 5/6 and 14 used in our study, and cytokeratin 5, p63 and P-cadherin in Gama et al.'s [34] study).

Stage and grade showed independent associations with survival in the multivariate regression (table 3), while molecular subtype grouping and histological type did not show associations. These data suggest that, at the moment, caution should be used when applying this classification system to the dog, in which the most useful information for prognosis is obtained from invasion and grade. However, when the dog is considered a spontaneous model of human breast cancer, similar tumour types should be compared between the two species. Because the morphological basis does not guarantee the grouping of biologically homogeneous tumours, application of a molecular-based system would improve the comparison of homogeneous groups. This study and that of Gama et al. [34] have demonstrated the existence of molecular-categorized groups, but the similarities between women and canine groups await confirmation in future investigations.

Application of the molecular-based classification system provides additional information on the different cell origin of basal-like and luminal-like tumours. Perou et al. [1] showed that basal-like tumours share some molecular features with myoepithelial cells which constitute the basal part of the normal epithelium, whereas luminal-like tumours have molecular features in common with normal luminal cells. In veterinary medicine the origin of the mesenchymal components in the so-called complex and mixed pattern of mammary tumours is still a matter of debate, and the myoepithelial cell is the most probable origin [35]. In our cases the basal-like group shared some characteristics with luminal-like A tumours (high percentage of grade 1) that are not known in human cancer and also conflict with the findings of Gama et al. [34]. This discrepancy may have two explanations. Firstly, the panel of antibodies and the criteria adopted to identify the positivity to basal differed in the two studies. It is known that characterization of the basal-like group is more difficult than that of HER2-overexpressing and luminal-like tumours, and for this reason several different panels and score methods have been proposed [36,37,9]. A second explanation is that some of our basal-like cases could be luminal-like tumours that were negative for ER and PR, but had positive myoepithelial cells that, in canine mammary tumours, are frequently expressed in the complex and mixed patterns. The criterion we adopted was at least 1% of cells positive to the basal markers in the invasive component, by virtue of the common myoepithelial proliferations that would induce a high number of false positive cases.

It is assumed that cytokeratin 5 is more sensitive than cytokeratin 14 as a basal marker because cytokeratin 5 could be expressed by bipotential progenitor cells as well as basal-like cells, whereas expression of cytokeratin 14 is limited to mature (basal) myoepithelial cells [9,36]. To better characterize the basal-like tumours and to avoid confusion with cases belonging to other groups, a panel of antibodies raised to several basal markers should be applied in canine mammary carcinomas.

## Conclusion

Application of a panel of antibodies, commonly used to characterize the molecular groups in human pathology disclosed three tumour groups (luminal-like A and B and basal-like) out of the five known (no ERB-B2 or normal-like cases were present in our samples). This finding strengthens the assumption that canine mammary tumours are a model of human breast cancer, but to make this comparison more reliable, homogeneous groups should be identified and compared. Canine malignant tumours appear to be a heterogeneous group of different molecular driven tumours which would benefit from a classification addressing molecular differences as in human medicine. However, the existence in the dog of all the groups proposed for human breast cancer and their diversity in biological behaviour await confirmation. Stage and grade showed independent associations with survival in the multivariate regression, while molecular subtype grouping and histological type did not show associations. These data suggest that, at the moment, caution should be used when applying this classification system to the dog, in which the most useful information for prognosis is obtained from invasion and grade.

## Competing interests

The authors declare that they have no competing interests.

## Authors' contributions

CB and GS conceived the study, participated in its design and coordination and drafted the manuscript. FS supervised the immunohistochemical stains and the scoring process. GS and GC performed the statistical analysis. All authors read and approved the final manuscript.
